# Does Maternal Obesity Affect Preterm Birth? Documentary Cohort Study of Preterm in Firstborns—Silesia (Poland)

**DOI:** 10.3390/children9071007

**Published:** 2022-07-06

**Authors:** Karolina Sobczyk, Tomasz Holecki, Joanna Woźniak-Holecka, Mateusz Grajek

**Affiliations:** 1 Department of Health Economics and Health Management, School of Health Sciences in Bytom, Medical University of Silesia in Katowice, 41902 Bytom, Poland; ksobczyk@sum.edu.pl (K.S.); tholecki@sum.edu.pl (T.H.); 2 Department of Health Promotion, School of Health Sciences in Bytom, Medical University of Silesia in Katowice, 41902 Bytom, Poland; jwozniak@sum.edu.pl; 3 Department of Public Health, School of Health Sciences in Bytom, Medical University of Silesia in Katowice, 41902 Bytom, Poland

**Keywords:** pregnancy, preterm birth, prematurity, obesity, overweight

## Abstract

In addition to low birth weight and intrauterine growth restriction, prematurity is a major problem in modern neonatology. The etiology of premature delivery is multifactorial, but maternal obesity has been indicated as an important risk factor for preterm birth. This study aimed to assess the relationship between early pregnancy body mass index (BMI) and the risk of preterm delivery according to gestational age. In the cohort of 2794 firstborns, preterm deliveries accounted for 9.1%. Of all deliveries, 16, 48, and 189 were classified as extremely preterm, very preterm, and moderately preterm deliveries, respectively. The risk of extremely, very, and moderately preterm deliveries increased with the increasing BMI, with the highest overweight and obesity-related risk for extremely and very preterm delivery. The rate of extremely and very preterm delivery among normal-weight women (BMI 18.5 ≤ 25) was 1.8%, while that among overweight and obese women (BMI ≥ 25) was 2.36%. The rate of all preterm deliveries (22 ≤ 37 weeks) was 8% for normal-weight women and 10.3% for overweight and obese women. Compared with normal-weight women, the adjusted odds ratio (95% CI) for preterm delivery in overweight and obese women was 1.33 (0.98–1.79). In Poland, being overweight and obese during pregnancy was associated with an increased risk of preterm delivery, especially extremely and very preterm delivery. This relationship should be assessed in other populations.

## 1. Introduction

Preterm birth is the leading cause of infant morbidity and mortality worldwide; hence, it is a significant public health concern. Therefore, PTB and the resulting prematurity of newborns are one of the major challenges for modern obstetrics and neonatology. Despite the development of medical technologies in recent years, preterm birth (PTB) is a leading problem faced by obstetricians and gynecologists [1]. The global rate of preterm births ranges between 5% and 18%, depending on the country, and despite many preventive interventions, an increased incidence of PTBs has been observed in most industrialized countries over the past two decades [2]. The growing number of medically induced preterm births and the growing prevalence of risk factors, such as advanced maternal age, is considered to be the main causes of this trend [3]. Globally, PTB is the main cause of perinatal mortality as well as neonatal morbidity and mortality [4]. It should also be emphasized that prematurity is associated with the risk of morbidity and mortality not only during the neonatal period but also during later stages of the child’s life, and it may contribute significantly to long-term disability in the child [1]. 

Preterm birth is defined by the World Health Organization (WHO) as birth after the completion of 22 weeks of gestation and before the completion of 37 weeks of gestation, regardless of birth weight [5]. According to the WHO estimates, there were approximately 15 million PTBs in 2010, accounting for 5–8% of all births worldwide [6]. The highest rates were reported in South Asia (13.3%) and Sub-Saharan Africa (12.3%), whereas the lowest rate was in the European region (8.6%) [6]. Preterm birth complications are the leading cause of death among children under 5 years of age, responsible for approximately 1 million deaths in 2015 [7]. In 2014, estimates from vital statistics data suggested that globally between 9% and 12% of births occur before 37 weeks of gestation, resulting in around 14.84 million preterm births a year [8]. The problem of preterm birth is also a leading problem faced by obstetricians and gynecologists in Poland. According to data from the Central Statistical Office, there were over 369,300 live births in Poland in 2020, including over 27,800 (7.5%) PTBs [9]. It should also be noted that in Poland, the percentage of cesarean sections is one of the highest in Europe, at 44%, and high BMI increases the risk of cesarean section [10,11]. In 2016, among adults in Poland, as many as 53% of women were overweight, and 23% of women were obese [10]. These are among the worst indicators in Europe. Projections are not optimistic—it is estimated that 26% of adult women in Poland will be obese in 2025 [10,12]. In contrast, in 2016, globally, 40% of women aged 18 years and over were overweight. Overall, about 15% of the world’s women population were obese in 2016 [13]. Because women are an integral part of the obese population, the impact of obesity on the health and wellbeing of mothers and their offspring is profound. Consequently, maternal obesity challenges modern perinatology [14]. Obesity’s etiology and repercussions in the setting of pregnancy are complex. Obese mothers require risk-adapted pregnancy treatment since obesity is linked to poor maternal and fetal outcomes [15]. To the authors’ knowledge, this is the first population-based study of the relationship between preterm birth risk and maternal obesity in Poland, including such a large study group. In Poland, there are no electronic registers for collecting data on the course of pregnancy, so it is not possible to conduct this kind of research using data from public statistics.

Growth in the number of PTBs has been observed over the last 30 years, which is associated mainly with the increased number of complicated pregnancies diagnosed between 34 and 36 weeks of gestation and the increasing tendency to consider planned births, which are regarded as safer than the continuation of complicated pregnancies. The increase in the number of PTBs in the last 10 years is strongly correlated with the higher number of patients with medical indications for the earlier termination of pregnancies and those giving birth after 35 years of age [16]. The cause of PTB is unknown in many cases [17,18,19]. 

Marchi et al. conducted quality assurance using the AMSTAR tool and data extraction steps in pairs. An analysis of the 22 review articles included revealed that gestational diabetes, pre-eclampsia, gestational hypertension, depression, instrumental and cesarean birth, and surgical site infection were more likely to occur in pregnant women with obesity than in women with a healthy weight [19]. Maternal obesity was also found to be associated with a greater risk of PTB, large-for-gestational-age babies, fetal defects, congenital anomalies, and perinatal death [20]. Similar conclusions were reported by Heslehurst [21] and Gould [22]. A meta-analysis showed a significant relationship between obesity and increased odds of cesarean and instrumental deliveries, preterm deliveries, hemorrhage, infection, a longer duration of hospital stay, and increased neonatal intensive care requirement [21]. Maternal obesity significantly contributes to poor prognoses for the mother and the baby during delivery and in the immediate post-partum period. Every initiative to decrease maternal obesity represents an important strategy for reducing PTB [22]. 

Therefore, considering the constant monitoring of the phenomenon and reports that do not unequivocally determine the impact of overweight and obesity on preterm birth, it seems valuable to continue to analyze the phenomenon. The main objective of this study was to assess the relationship between early pregnancy BMI and the risk of preterm delivery according to gestational age. The study hypothesized that maternal overweight and obesity and associated elevated anthropometric indices influence preterm delivery. In addition, the publication includes a secondary objective, such as indicating the relationship between abnormal BMI and age and selected socioeconomic characteristics of the pregnant population studied.

## 2. Materials and Methods

### 2.1. Background

A medical record-based cohort study of firstborns was conducted among women who gave birth between 2018 and 2020. Medical records of pregnancy and delivery were analyzed. The primary research tool was a database prepared for the study, in which information extracted from medical records was recorded. The data were obtained by applicable law as a result of consent to conduct the study. In light of the Polish law (Act of 5 December 1996, Ustawa o zawodach lekarza i lekarz-dentysty), work based on medical records is not subject to the decision on medical experimentation, which is also in accordance with the Helsinki declaration and statement of the Bioethics Committee of the Medical University of Silesia in Katowice (no. KNW/0022/KB/54-1/13). Maternal height and maternal weight were measured by medical staff at the beginning of pregnancy (these data were taken from medical records for our study). Patients gave their consent to participate in the study, including the use of data collected in medical records and the publication of the results.

### 2.2. Eligibility Criteria

The inclusion criterion was information in the records about a university hospital stay (the Medical University of Silesia in Katowice) related to a firstborn live birth (birth after the 22nd week of gestation) and availability of early pregnancy BMI. Firstborns were selected because there is a risk of bias in the results of the statistical analysis resulting from the fact that women, in some cases, do not return to their pre-pregnancy weight. Exclusion criteria were multiple births, pregnancy loss (delivery before 22 weeks of gestation), general or pregnancy-related diseases, stillbirth, and neonatal death before the end of hospitalization. Based on the exclusion criteria, 328 women were not eligible for inclusion in the study; another 27 refused to participate in the study. The main reason for exclusion from the study was multiple births (76.8% of these 328 women). Therefore, 2794 patients were included in the study. The criterion of firstborns was adopted to exclude other confounders to the study, such as the possibility of physiological and behavioral changes after a previous pregnancy (overweight and obesity from previously delivered children).

### 2.3. Characteristics of the Study Group

The mean age of the patients was 29.3 years (median 29 years). Of all deliveries, 1534 were vaginal deliveries (53.4%), and 1338 (47.6%) were cesarean deliveries. Preterm deliveries accounted for 9.1% (253 cases) of all deliveries. The high percentage of cesarean sections was due to the fact that the study participants were recruited from a tertiary referral center (dedicated to the care of the most severe pregnancy pathology). 

### 2.4. Basic Calculations

The main measure was the risk of preterm delivery (extremely, 22–27 weeks; very, 28–31 weeks; and moderately, 32–37 weeks). BMI was calculated from height and weight data at the first prenatal visit between 8 and 12 weeks of gestation. Maternal height and maternal weight were measured with light indoor clothing. Based on BMI, women were classified as underweight (BMI < 18.5), normal (18.5 ≤ 25), overweight (25 ≤ 30), or obese (≥30).

### 2.5. Statistical Analyses

Data were statistically analyzed using R environment and Statistica v12 software (StatSoft). The Kolmogorov-Smirnov test was used to verify the distribution of quantitative variables. The null hypothesis of normality of distribution was rejected for all variables. The distribution of qualitative and semiquantitative variables was analyzed using frequencies of individual values, and the χ^2^ test of independence was used to determine the frequency of qualitative variables in each group. Cross-tabulations, number of cases, percentages, and significance (α) levels were also used to analyze the data. A logistic regression model was used to calculate the probability of events using odds ratios (OR) with 95% confidence intervals and corresponding significance levels. Predictive factors were BMI categories and potential confounders (maternal age, education level, and work situation). The variables were categorized as shown in Table 1. A statistical significance level of α = 0.05 and a probability value (*p*-value ≤ 0.05) were used for the calculations.

## 3. Results

The study included data on 2794 live firstborns. The mean BMI in the study group was 23.1. Abnormal body weight was reported in 32% of women, underweight in 8.4% of women, overweight in 15.7% of women, and obesity in 7.9% of women (Table 1). Data analysis showed statistically significant higher rates of overweight and obesity in the sample (*p* < 0.001). The problem of overweight and obesity was shown to be statistically significant in older age groups. The positive correlation between age and the quantitative variable BMI was confirmed by the Spearman correlation (*p* < 0.001; Rs = 0.09) and the Kruskal-Wallis test (*p* < 0.001; H = 22.9). According to the results of the logistic regression model, the risk of overweight and obesity was 41%, 54%, and 49% higher in patients aged 25–29, 30–34, and ≥35 years, respectively, compared to pregnant women <25 years of age. 

The highest rates of overweight and obesity were reported for married women with secondary or vocational education and those in lower-income groups (*p* < 0.001). The results were partially confirmed by the results of the logistic regression model, which, although not statistically significant, showed a 15% higher risk of overweight and obesity in married women than in unmarried women (OR: 1.15; CI: 0.93–1.42; *p* = 0.18). The risk of overweight and obesity was higher in groups with a low income per person in the family (OR: 1.5; CI: 1.24–1.8; *p* < 0.001) (Table 2).

In the cohort of 2794 firstborns, preterm deliveries accounted for 9.1%. Of all deliveries, 16, 48, and 189 were classified as extremely preterm, very preterm, and moderately preterm deliveries, respectively (Table 3). The study showed that PTBs were significantly more common in overweight and obese patients (*p* < 0.001) compared to normal-weight women. Risk estimates were adjusted for maternal age, education level, and professional situation. The logistic regression model showed a 33% higher risk of preterm birth for overweight or obese patients (OR: 1.33; CI: 0.98–1.79; *p* < 0.05) compared to normal-weight women. The risk of extremely preterm, very preterm, and moderately preterm deliveries increased with the increasing BMI, with the highest being overweight and obesity-related risk for extremely and very preterm delivery. The rate of extremely and very preterm delivery among normal-weight women (BMI 18.5 ≤ 25) was 1.8%, while that among overweight and obese women (BMI ≥ 25) was 2.36%. The rate of all preterm deliveries (22 ≤ 37 weeks) was 8% for normal-weight women and 10.3% for overweight and obese women (Table 3 and Table 4).

## 4. Discussion

Our study showed an increased risk of PTB among patients with an abnormal BMI. Many authors reported the negative impact of extreme BMI values on PTBs [23,24,25,26,27,28,29]. Overweight and obesity are currently considered a global issue, and the number of individuals with BMI ≥ 30 has doubled since 1980. As estimated by the WHO, 1.9 billion adults are overweight, and 600 million are obese [30,31]. There has also been a growing trend in the prevalence of overweight and obesity in Poland. According to the data of the Central Statistical Office for 2020, 30% and 15.5% of women in the general Polish population are overweight and obese, respectively [32,33]. Although precise data on the incidence of abnormal body weight among women of reproductive age are missing, our findings, as well as those reported by other authors, indicate that overweight and obesity are also major problems in this group. This study identified 16.1% of overweight and 8.1% of obese women in early pregnancy in the analyzed population, which corresponds to the data obtained, for example, in the Dutch (18.7% and 7%) [29], Russian (18.3% and 7.1%) [34], and Chinese population (18.3% and 6.8%) [35]. Higher rates were reported by researchers in Scotland (20.8% and 8.5%) [36] and the United States (20.3% and 15.5%) [37]. This analysis also showed that age >30 years and low income are risk factors for overweight and obesity among women of childbearing potential. All the aforementioned factors were associated with a higher risk of overweight and obesity in populations investigated in other studies [34,36,37,38].

The findings of this study showed that abnormal body weight might be one of the important risk factors for preterm birth. Cnattingius et al. [36] also showed a significant relationship between maternal BMI and PTBs in their study. Although the rates of births before 36 completed weeks of gestation in their cohort study were lower (5%) compared to the findings of this study (10.6%), which may be due mainly to the prevalence of PTBs in the last 25 years (Cnattingius et al. 1992–2010) and in addition this study was limited to a tertiary referral center with the percentage of PTBs being similar in different BMI groups. Cnattingius et al. analyzed a sample of more than 1.5 million singleton births in Sweden, showing 1.4 times and 1.2 times higher incidence of PTBs among underweight and obese mothers, respectively. Similar results were obtained in this study with 1.7-fold and 1.2-fold higher rates of PTBs in groups of patients with BMI < 18.5 and BMI ≥ 30, respectively. Furthermore, the above-cited Swedish cohort study showed that older women (>35 years of age), multifetal, as well as women with pregnancy-induced hypertension and/or gestational diabetes, accounted for the majority of overweight or obese patients. The findings of this study confirm these conclusions. 

In a study in Russia, Sharashova et al. [34] also showed a relationship between maternal BMI and PTBs, reporting findings similar to those reported in this study. The authors concluded, based on their analysis of medical records of more than 29,000 underweight and obese patients, that the risk of PTB in these two groups is 25% and 31% higher, respectively, compared with their normal body weight counterparts. In this study, this risk was 33% higher for overweight and obese women. In a study conducted in California, Kosa et al. [35] analyzed more than 1000 births and showed a 30% increase in the risk of PTB among obese women, which is consistent with this study’s findings. Furthermore, it was shown that the risk of PTB increases with increasing BMI among women with BMI > 24 and that patients with BMI = 29 are at a 40% higher risk compared to those with BMI = 24. 

A Polish study conducted by Mikołajek-Bedner et al. among 150 pregnant women in Szczecin also showed higher rates of birth before the completion of 36 weeks of gestation in the group of obese patients [38]. Slack et al. observed that there was an association between the absolute risk of extreme, very, and moderate preterm birth and BMI category, with the greatest effect size being extreme preterm. The absolute risk of post-term birth increased monotonically as the BMI category increased. The largest effect sizes were observed for class IIIb obesity and extreme preterm birth (adjusted OR 2.80, 95% CI 1.31–5.98) [39].

Infections and inflammation are increasingly seen as important obesity-related elements of metabolic syndrome. Research on gene expression in the population of obese pregnant women has shown increased levels of pro-inflammatory cytokines as a result of their release by adipose tissue [40]. Furthermore, these compounds, along with macrophages, were found in the placenta of obese pregnant women. Obese women’s chronic inflammation before pregnancy sets off a chain of events that results in an inflammatory in utero environment. Moreover, the inflammatory environment in which the fetus grows has negative repercussions for obesity and insulin resistance syndrome metabolic programming [41]. These observations partially account for the increased risk of preterm birth among women with high BMI. It should also be emphasized that overweight and obesity are important risk factors for pregnancy complications, such as hypertension or gestational diabetes; therefore, they may indirectly contribute to an increased risk of birth before completing 36 weeks of gestation [37,38,42,43,44,45,46,47]. 

In scientific studies, among the numerous risk factors of PTB, those belonging to the group of socioeconomic factors are also mentioned, including mainly low education, low socioeconomic status, and single marital status [48,49]. This fact confirms that the causes of preterm deliveries are complex, and the problem should continue to be addressed by further researchers.

### Strengths and Limitations

An undeniable strength of conducting the study is the large study group of 2794 firstborns, which allows the results to be related to the general population. The results of the study provide a nucleus for further research into the nature of preterm births in women with elevated BMI. In the future, the authors plan to implement an intervention program aimed at promoting healthy eating habits and introducing physical activity among women planning pregnancies as basic elements of procreative health promotion.

The authors also note the weaknesses of the study. The study takes into account only data from medical records, and one should have confidence that this was conducted in a reliable manner. Therefore, an information reliability test was introduced in the study, which indicated that 93% contained data that had some repeatability. The main limitations of the study include the lack of an integrated database of such information in Poland, such as in the UK or Scandinavian countries. Another limitation is the minimalism of medical records maintained during hospitalization, focused mainly on the information necessary to bill the services to the public payer. As a consequence, this makes it impossible, for example, to distinguish between different types of preterm birth or to identify women with a potential earlier diagnosis of pathology. There appear to be additional variables that should be considered in further research (e.g., ethnicity and gestational weight gain). It is worth emphasizing that the results of the study should be generalized to the general population with great care due to the sample size and the fact of primacy. 

## 5. Conclusions

In summary, maternal overweight and obesity during pregnancy were associated with an increased risk of preterm delivery, especially extremely and very preterm delivery among Polish women. These correlations should be assessed in other populations. Considering the far-reaching global spread of prematurity and the resulting mortality among newborns, infants, and children ≤5 years of age, integrated actions for the prevention of preterm births are needed. These interventions should be addressed not only in pregnant women at risk of PTB but also in all pregnant women and women of childbearing age. In order to ensure the comprehensiveness of the measures taken, they should be complemented by high-quality procedures for the health care of premature infants.

The results of the study stand in a different position from the research evidence presented in the discussion. Some studies confirm that increased maternal weight has an effect on preterm birth others deny this and even report that being overweight may be a protective factor. Thus, further monitoring of this phenomenon seems warranted.

## Figures and Tables

**Table 1:** Maternal characteristics and rates of preterm firstborns.

		Weeks Gestation, No. (%)*							
	No. of Women (n = 2794)	22–27 (n = 16)		28–31 (n = 48)		32 ≤ 37 (n = 189)		≥37 (n = 2541)	
Body mass index									
<18.5	241	5	(31.3)	9	(18.8)	20	(10.6)	207	(8.1)
18.5 ≤ 25	1876	7	(43.8)	27	(56.2)	116	(61.4)	1726	(67.9)
25 ≤ 30	450	3	(18.7)	7	(14.6)	41	(21.7)	400	(15.8)
≥30	226	1	(6.2)	5	(10.4)	12	(6.3)	208	(8.2)
Age, y									
<25	556	1	(6.2)	4	(8.3)	26	(13.8)	449	(17.7)
25–29	986	5	(31.3)	9	(18.8)	38	(20.1)	557	(21.9)
30–34	875	4	(25.0)	14	(33.3)	53	(28.0)	698	(27.5)
≥35	455	6	(37.5)	21	(39.6)	72	(38.1)	837	(32.9)
Education									
primary	204	5	(31.3)	14	(33.3)	44	(23.3)	524	(20.6)
vocational	339	4	(25.0)	13	(27.1)	47	(24.9)	598	(23.5)
secondary	961	4	(25.0)	9	(18.8)	38	(20.1)	642	(25.3)
higher	1271	3	(18.7)	10	(20.8)	43	(22.8)	716	(28.2)
data missing	19	0	(0)	0	(0)	1	(8.9)	61	(2.4)
Professional situation									
unemployed	605	10	(62.5)	26	(54.2)	105	(47.2)	1086	(42.7)
employed	2157	6	(37.5)	22	(45.8)	83	(43.9)	1287	(50.6)
data missing	32	0	(0)	0	(0)	1	(8.9)	168	(6.6)

**Table 2:** Early pregnancy BMI depending on selected socioeconomic factors.

Marital Status	BMI Categories, No. (%)								*p*-Value for Test χ^2^
	<18.5		18.5 ≤ 25		25 ≤ 30		≥30		
marriage (n = 2083)	138	(6.6)	1387	(66.6)	334	(16.0)	180	(8.6)	***p* < 0.001**
informal relationship (n = 617)	67	(10.9)	394	(63.9)	94	(15.2)	36	(5.8)	
single state (n = 145)	33	(22.8)	82	(56.6)	18	(12.4)	5	(3.4)	
Education									
primary (n = 204)	35	(17.2)	128	(62.7)	19	(9.3)	13	(6.4)	***p* < 0.001**
vocational (n = 339)	40	(11.8)	194	(57.2)	54	(15.9)	32	(9.4)	
secondary (n = 961)	80	(8.3)	602	(62.6)	160	(16.6)	97	(10.1)	
higher (n = 1271)	78	(6.1)	893	(70.2)	206	(16.2)	73	(5.7)	
Professional situation									
unemployed (n = 605)	81	(13.4)	358	(59.2)	99	(16.4)	45	(7.4)	***p* < 0.001**
employed (n = 2157)	152	(7.0)	1455	(67.5)	332	(15.4)	169	(7.8)	
Net income/person in the family (€)									
<120 n = 296	44	(14.9)	163	(55.1)	47	(15.9)	26	(8.8)	
120–240 n = 691	75	(10.9)	405	(58.6)	123	(17.8)	66	(9.6)	
240–480 n = 1042	79	(7.6)	697	(66.9)	163	(15.6)	81	(7.8)	***p* < 0.001**
480–720 n = 463	25	(5.4)	344	(74.3)	62	(13.4)	25	(5.4)	
≥720 n = 264	11	(4.2)	197	(74.6)	33	(12.5)	3917	(6.4)	

**Table 3:** The rates of preterm birth depending on early pregnancy BMI.

BMI Categories	Preterm Birth (n = 2794), No. (%)				*p*-Value for Test χ^2^
	Yes		No		
<18.5 (n = 241)	34	(14.1)	207	(86.3)	***p* < 0.001**
18.5 ≤ 25 (n = 1876)	150	(8.0)	1721	(92.0)	
25 ≤ 30 (n = 450)	47	(10.6)	402	(89.4)	
≥30 (n = 226)	22	(9.7)	204	(90.3)	

**Table 4:** Logistic regression results for maternal BMI in early pregnancy and risks of preterm delivery (n = 2 794).

	BMI Categories			*p*-Value for Test χ^2^
	<18.5	18.5 ≤ 25	≥25	
Preterm delivery *				***p* < 0.05**
No. (%)	34 (13.4)	150 (59.3)	69 (27.3)	
Adjusted OR (95% Cl) *	1.17 (0.9–1.52)	1 [reference]	1.94 (1.56–2.42)	
Extremely and very preterm delivery *				
No. (%)	14 (5.5)	34 (13.4)	16 (6.3)	
Adjusted OR (95% Cl) *	1.04 (0.62–1.73)	1 [reference]	2.36 (1.87–2.98)	
Moderately preterm delivery *				
No. (%)	20 (7.9)	116 (45.9)	53 (21.0)	
Adjusted OR (95% Cl) *	1.26 (1.14–1.39)	1 [reference]	1.81 (1.58–2.07)	

* variables used: BMI Categories, preterm delivery included extremely and very preterm delivery and moderately preterm delivery.
